# Supplementary material for: Factors associated with health worker adoption of facial and environmental hygiene promotion in the ‘SAFE strategy’ for trachoma elimination in Western Province, Zambia
Source: PLoS Negl Trop Dis. 2025 Sep 8;19(9):e0013483. doi: 10.1371/journal.pntd.0013483 (PMC12431646; doi:10.1371/journal.pntd.0013483)
Supplement: Attachment — Submitted filename: PNTD-D-25-00493.pdf [file pntd.0013483.s002.pdf]

PLOS Neglected Tropical Diseases

Determinants of adoption of facial and environmental hygiene promotion in the ‘SAFE’ strategy for trachoma control in Western Province, Zambia – a cross-sectional study

--Manuscript Draft--

**Manuscript Number:**	PNTD-D-25-00493
**Full Title:**	Determinants of adoption of facial and environmental hygiene promotion in the ‘SAFE’ strategy for trachoma control in Western Province, Zambia – a cross-sectional study
**Short Title:**	Adoption of facial and environmental hygiene promotion for trachoma control
**Article Type:**	Research Article
**Keywords:**	Determinants; Adoption; Facial and environmental hygiene promotion; SAFE; Trachoma; Western Province; Zambia
**Abstract:**	Background<br><br>Trachoma is responsible for the blindness or visual impairment of about 1.9 million people and causes about 1.4% of all blindness worldwide. The SAFE (Surgery, Antibiotics, Facial Cleanliness and Environmental Improvement) strategy is recommended for control of trachoma. In many lowincome settings, interventions particularly for facial cleanliness and environmental improvement have been sub-optimally adopted. This study sought to establish the level and determinants of adoption of the intervention, facial and environmental hygiene promotion among health care workers in Western Province, Zambia.<br><br>Methodology/principal findings<br><br>This was a cross-sectional study involving twenty-four health facilities selected from three districts using stratified random sampling. From these, 388 health workers (Environmental health officers, community health assistants and community health workers) were randomly selected. Multiple logistic regression was used to identify the determinants of adoption.<br>Adoption of the intervention was low at 47.68%. The most widely adopted core intervention strategy was community demonstrations for correct face and handwashing. Working in districts, Kaoma (AOR = 0.43, 95% CI = [0.24, 0.76]) and Shangombo (AOR = 0.38, 95% CI = [0.17, 0.83]) were associated with lower odds of adoption. Having readily available transport (AOR = 3.04, 95% CI = [1.38, 6.70]), perceiving the intervention as relevant (AOR = 8.15, 95% CI = [4.59, 14.46]), having been trained in F and E (AOR = 2.19, 95% = [1.26, 3.79]) and availability of tools and information, education and communication material (AOR = 2.94, 95% CI = [1.65, 5.25]) were associated with higher odds of adoption.<br><br>Conclusion/significance<br><br>There was low adoption of facial and environmental hygiene promotion influenced by training, transport, tools and IEC material availability and perceived relevance and complexity of the intervention. Strategies to increase the adoption process should be tailored according to these factors. Differences in adoption between districts suggests the need for further research to identify determinants of adoption in the different settings
**Additional Information:**	
**Question**	**Response**
**Government Employee**<br><br>Are you or any of the contributing authors an employee of the United States government?	No - No authors are employees of the U.S. government.

Powered by Editorial Manager® and ProduXion Manager® from Aries Systems Corporation

Manuscripts authored by one or more US Government employees are not copyrighted, but are licensed under a CC0 Public Domain Dedication, which allows unlimited distribution and reuse of the article for any lawful purpose. This is a legal requirement for US Government employees.<br><br>This will be typeset if the manuscript is accepted for publication.	
**Financial Disclosure**<br><br>Enter a financial disclosure statement that describes the sources of funding for the work included in this submission. Review the submission guidelines for detailed requirements. View published research articles from *PLOS Neglected Tropical Diseases* for specific examples.<br><br>This statement is required for submission and **will appear in the published article** if the submission is accepted. Please make sure it is accurate.<br><br>**Funded studies**<br>Enter a statement with the following details:<br>• Initials of the authors who received each award<br>• Grant numbers awarded to each author<br>• The full name of each funder<br>• URL of each funder website<br>• Did the sponsors or funders play any role in the study design, data collection and analysis, decision to publish, or preparation of the manuscript?<br><br>Did you receive funding for this work?	The author(s) received no specific funding for this work.
**Competing Interests**<br><br>On behalf of all authors, disclose any competing interests that could be perceived to bias this work.	The authors have declared that no competing interests exist

This statement will be typeset if the manuscript is accepted for publication.<br><br>Review the instructions link below and PLOS NTDs' competing interests policy to determine what information must be disclosed at submission.	
**Data Availability**<br><br>Provide a **Data Availability Statement** in the box below. This statement should detail where the data used in this submission can be accessed. This statement will be typeset if the manuscript is accepted for publication.<br><br>Before publication, authors are required to make all data underlying their findings fully available, without restriction. Review our PLOS Data Policy page for detailed information on this policy. Instructions for writing your Data Availability statement can be accessed via the Instructions link below.	All relevant data are within the manuscript and its Supporting Information files

Manuscript Click here to access/download;Manuscript;Manuscript March 2025_Martha Kasongo.pdf

Determinants of adoption of facial and environmental hygiene promotion in the 'SAFE' strategy for trachoma control in Western Province, Zambia – a cross-sectional study

Martha Kasongo[1], Choolwe Jacobs[1], Silumbwe Adam[2], Patricia Maritim[3], Joseph Mumba Zulu[3], Hikabasa Halwindi[4]

Author Affiliations

[1] Department of Epidemiology and Biostatistics, School of Public Health, University of Zambia, Lusaka, Zambia

[2] Department of Health Policy and Management, School of Public Health, University of Zambia, Lusaka, Zambia

[3] Department of Health Promotion and Education, School of Public Health, University of Zambia, Lusaka, Zambia

[4] Department of Community and Family Medicine, School of Public Health, University of Zambia, Lusaka, Zambia

Corresponding author

Email: mkasongo10@gmail.com (MK)

Abstract

Background

Trachoma is responsible for the blindness or visual impairment of about 1.9 million people and causes about 1.4% of all blindness worldwide. The SAFE (Surgery, Antibiotics, Facial Cleanliness and Environmental Improvement) strategy is recommended for control of trachoma. In many low-income settings, interventions particularly for facial cleanliness and environmental improvement have been sub-optimally adopted. This study sought to establish the level and determinants of adoption of the intervention, facial and environmental hygiene promotion among health care workers in Western Province, Zambia.

Methodology/principal findings

This was a cross-sectional study involving twenty-four health facilities selected from three districts using stratified random sampling. From these, 388 health workers (Environmental health officers, community health assistants and community health workers) were randomly selected. Multiple logistic regression was used to identify the determinants of adoption.

Adoption of the intervention was low at 47.68%. The most widely adopted core intervention strategy was community demonstrations for correct face and handwashing. Working in districts, Kaoma (AOR = 0.43, 95% CI = [0.24, 0.76]) and Shangombo (AOR = 0.38, 95% CI = [0.17, 0.83]) were associated with lower odds of adoption. Having readily available transport (AOR = 3.04, 95% CI = [1.38, 6.70]), perceiving the intervention as relevant (AOR = 8.15, 95% CI = [4.59, 14.46]), having been trained in F and E (AOR = 2.19, 95% = [1.26, 3.79]) and availability of tools and information, education and communication material (AOR = 2.94, 95% CI = [1.65, 5.25]) were associated with higher odds of adoption.

Conclusion/significance

There was low adoption of facial and environmental hygiene promotion influenced by training, transport, tools and IEC material availability and perceived relevance and complexity of the intervention. Strategies to increase the adoption process should be tailored according to these factors. Differences in adoption between districts suggests the need for further research to identify determinants of adoption in the different settings

Keywords: Determinants, Adoption, Facial and environmental hygiene promotion, SAFE, Trachoma, Western Province, Zambia.

Author Summary

Trachoma remains a public health problem in Zambia and other countries. WHO recommends the surgery, antibiotics, facial cleanliness and environmental improvements (SAFE strategy) for control of trachoma in endemic communities. Studies have shown that facial and environmental hygiene promotion intervention of this strategy has not been adequately adopted in many places and the determinants of adoption are unclear. We determined the level of adoption of facial and environmental hygiene promotion among health workers and identified the determinants of adoption of the intervention in three districts in Western Province, Zambia. We found that adoption of the intervention was generally low (47.68%) and was highest in Kalabo district compared to the other districts included in the study. The most widely adopted core component of the intervention was community demonstrations for correct face and hand washing methods. Participants who had available transport, who perceived the intervention as relevant for trachoma prevention, who had received training in the intervention and those who had tools and IEC material were more likely to adopt the intervention compared to those who did not have these characteristics. Trachoma

control program officers and stakeholders should tailor strategies for facial and environmental hygiene promotion according to these factors.

Introduction

Trachoma mostly affects communities with inadequate access to water and sanitation [1]. Globally, there are over 40 million active cases of trachoma of which 8.2 million have trichiasis, the blinding stage. Previous surveys in Zambia found that 16 districts have trachoma prevalence > 5% and nearly 1.5 million people needed interventions with A, F and E components of the Surgery, Antibiotics, Facial Cleanliness and Environmental improvement (SAFE) strategy. These surveys showed that Western Province had the highest prevalence of trachoma with some districts having as high as 17% prevalence of trachomatous follicular in children aged 1-9 years old [2].

The World Health Organization (WHO) recommends the SAFE strategy for the control and elimination of trachoma [3]. In this strategy, surgery is used to stop trichiasis cases from leading to blindness, antibiotics are used to treat active infections and prevent further transmission and facial cleanliness and environmental improvements to prevent transmission by flies (WHO, 2021). Improvements in facial cleanliness and environmental hygiene have led to reduction of trachoma prevalence and elimination of trachoma in some countries [4]. Facial and environmental hygiene promotion is mainly done by health workers in collaboration with community health workers [5]. In Zambia, the core components of facial and environmental hygiene promotion include community sensitization using information, education and communication (IEC) material and demonstrations for correct face and hand washing methods [6]. Other intervention components include holding /attending quarterly stakeholder consultation meetings, promotion of face washing

and handwashing of children in schools and engagement of community leaders to champion change in their communities [6].

Despite the importance of facial and environmental hygiene promotion, there is evidence that the intervention has not been implemented adequately and this could explain the slow progress towards elimination of trachoma in some endemic countries [7]. For example, a study in Ethiopia found that there was lack of implementation of facial and environmental hygiene promotion activities as only 44% and 24% of study settings adopted and implemented facial and environmental hygiene promotion activities respectively [8]. Similarly, the WHO highlights that inadequate adoption and implementation of facial cleanliness and environmental improvement interventions is one of the major factors driving persistent trachoma in countries such as Zambia [7].

In previous studies, adoption of facial and environmental hygiene promotion has been reported to be affected by lack of prioritization of the intervention activities, inadequate staffing in health facilities and lack of resources [9, 10]. There is still very little documentation of the determinants of adoption of facial and environmental hygiene promotion activities as few studies have focused on this topic especially in low-income settings like Zambia. Limited studies on the 'SAFE' strategy have concentrated on the facial cleanliness and environmental improvement components [11]. Understanding the determinants of adoption of facial and environmental hygiene promotion can help trachoma prevention program implementers identify possible solutions to enhance adoption of the intervention and develop impactful implementation strategies, which can in turn enhance trachoma control [11]. This study therefore sought to establish the level and determinants of adoption of facial and environmental hygiene promotion among health care workers in Western Province, Zambia. The results will inform trachoma program officers and stakeholders of the level

of adoption of facial and environmental hygiene promotion and guide the selection of strategies to improve adoption of the intervention through the identified determinants of adoption

Methods

Conceptual or theoretical framework

This study employed the RE-AIM/PRISM framework (Reach, Effectiveness, Adoption, Implementation and Maintenance framework and Pragmatic Robust, Implementation and Sustainability Model), an evaluation framework which was developed to enhance the evaluation of community based health promotion and public health interventions [12]. This framework conceptualizes the public health impact of an intervention as a function of 5 factors, reach, effectiveness, adoption, implementation and maintenance. These RE-AIM outcomes are influenced by contextual and explanatory factors such as characteristics of organization recipients e.g. availability of transport, IEC material and skills that can affect the implementation of an intervention [12]. The study used the RE-AIM framework to guide the assessment of adoption and changing internal context domain of PRISM to guide the selection and assessment of determinants of adoption. According to the RE-AIM framework, adoption refers to the proportion and representativeness of settings such as worksites, health departments or communities that adopt a given intervention. In this study, Adoption was measured in terms of what percent of workers participated in facial and environmental hygiene promotion. Within the framework, we focused more on adoption as it is one of the understudied areas in this field.

Study design

This was a cross-sectional study which was designed to determine the extent of adoption of facial and environmental hygiene promotion and identify the determinants of adoption among health care

workers. This is a type of observational study design in which the investigator measures the outcome and exposure in the study participants at the same time. This study design was ideal for determining the proportion of health workers who had adopted facial and environmental hygiene promotion while allowing the identification of the determinants of adoption of the intervention [13].

Study setting

This study was conducted in Shangombo, Kalabo and Kaoma districts in Western Province of Zambia in the months of March and April 2023. Seven districts were implementing the full ‘SAFE’ strategy in Western Province at the time of the study. However, only three districts met the inclusion criteria of having commenced implementation of the ‘SAFE’ strategy in 2013 and having a trachoma prevalence greater than 10% in children aged 1-9 years old as of 2018 [2, 6]. At the time of the study, Western Province had a total of 307 health facilities that were offering primary health care services. Of these facilities, approximately 52 were distributed in the study districts. Among other primary health care services, these primary health care facilities provided opportunities for preventive health interventions such as health education. Data was collected in primary health care facilities including urban health centres, rural health centres and health posts in the three districts.

Sampling and participant recruitment

The study population included environmental health technologists, community health assistants and community health workers. In the three districts (Kaoma, Kalabo and Shangombo), this population was estimated at 564 health workers (512 community health workers and 52

environmental health technologists and community health assistants) from 52 health facilities (Provincial Health Office, unpublished, 2022). The sample size was calculated using Cochran's equation, $n \geq (Z^2 p [1-p])/e^2$ where, n is the sample size, z is the selected critical value of desired confidence level, p is the estimated proportion of an attribute that is present in the population and e is the desired level of precision. Using 5% standard error and a 40% estimated proportion of health workers who adopted the intervention [14], a sample of 384 workers was calculated. However, a sample size of 390 was used to allow a 2% non-response rate. Stratified random sampling method was used to select participants. Workers were eligible to participate in the study if they had worked in their position for not less than one year. Participants were identified from a sampling frame of facility health workers which was obtained on request from the health facility in-charge officers. Using probability proportional to size, a representative sample size of health facilities and individual participants in each district was estimated and potential participants were then systematically selected from the sampling frame and invited to participate in the study via phone.

Data collection

Data was collected using a structured questionnaire with mostly closed ended questions. The questionnaire was prepared in English and translation to the local language, Lozi was done verbally during data collection whenever need arose. An online tool, Kobo Collect was used for data collection. Using the questionnaire, we collected data on the dependent variable, adoption of facial and environmental hygiene promotion and information on the potential internal contextual factors (independent variables) affecting the adoption of the intervention. The independent variables included socio-demographic factors (age, sex, level of education), position held, facility type, length of service, training, availability of tools and IEC material, availability of transport,

perceived intervention complexity, perceived intervention relevance and attitude towards facial and environmental hygiene promotion. Adoption was measured on a binary scale and all the independent variables were measured on the nominal scale except for age and length of service which were continuous variables. Healthcare workers were considered to have adopted facial and environmental hygiene promotion if they had conducted or taken part in community distribution of information, education and communication (I, E and C) materials or community demonstrations of correct hand and face washing methods or both in the past six months. Quantitative data was collected by the principal investigator with the help of 3 research assistants and the data was collected over a period of one month.

Data analysis

During data analysis, the distributions of continuous variables was checked for normality using a histogram with a super imposed normal curve. Both the continuous variables, age and length of service were not normally distributed and therefore, we reported the median and inter-quartile range summary statistics. To test any independent associations between categorical variables, Pearson's chi square test was used upon checking for assumptions. Otherwise, Fisher's exact test was used. The non-parametric, Wilcoxon rank sum test was used to check for associations between the outcome variable and continuous variables, age and length of service.

An investigator led backward stepwise multivariable logistic regression was used to identify the internal contextual determinants of adoption of facial and environmental hygiene promotion. The multiple logistic regression was selected as the best regression method for this study because the outcome variable, adoption was a binary outcome. Selection of the best model of factors was performed using the likelihood ratio test after estimation of the nested models by adding and eliminating variables one at a time. We reported robust estimates of odds ratios and confidence

intervals at both binary and multivariable logistic regression to counter any possible clustering effects. Data was analyzed using STATA software version 15.0 SE (Stata Corporation, College Station, TX, USA).

Ethical considerations

This study did not involve child participants. The study participants were health workers, all aged 18 years and older. All participants provided formal written informed consent in their preferred language (Lozi or English). The University of Zambia Biomedical Research Ethics Committee (REF.No. 3469-2022) and the National Health Research Authority approved this study and permission was obtained from provincial health office, district health offices as well as health facility in-charges. Confidentiality and anonymity were maintained by ensuring that the data collected did not contain personal identifiers and the data was stored on password protected gadgets.

Results

Characteristics of the participants

A total of 390 eligible health workers from 24 health facilities were sampled. Out of these, a total of 388 questionnaires were analyzed and two were disqualified due to incomplete information. Of the 388, just above half were females, 50.77% (197/388). The median age of the participants was 40 years (IQR: 34 – 50). Most of the respondents, 65.72% (255/388) had attained secondary education as their highest level of education. The majority, 68.56% (266/388) were community health workers and a small proportion, 4.64% (18/388) were environmental health technologists or officers. A larger proportion of participants, 34.79% (135/388) were from health posts. Of the

background characteristics, only district, supervisor position and transport availability were associated with the outcome. Sex, age, length of service, education and position of respondent were not (Table 1a).

Table 1a. Characteristics of the health care workers; Western Province, April 2023 (N=388)

**Factor**	**Frequency (%)**	Adopted F and E hygiene promotion n (%)	Did not adopt F and E hygiene promotion n (%)	P-values (Chi-square/Fisher's Exact/ Wilcoxon Rank sum)
**Sex**				
Male	197 (50.77)	91 (47.64)	100 (52.36)	0.9896^{c}
Female	191 (49.23)	94 (47.72)	103 (52.28)	
**Age (Median, IQR)**	40 (34, 50)	41 (34, 50)	40 (34,49)	0.687^{wr}
**Service length (Median, IQR)**	6 (3.5, 10)	6 (3, 10)	7 (4,11)	0.271^{wr}
**Education**				
No Education	6 (1.56)	3 (50.00)	3 (50.00)	0.185 ^{f}
Primary Level	91 (23.45)	39 (42.86)	52 (57.14)	
Secondary Level	255 (65.72)	120 (47.06)	135 (52.94)	
Tertiary Level	36(9.28)	23 (63.89)	13 (36.11)	
**District**				
Kalabo	163 (42.01)	96 (58.90)	67 (41.10)	**0.001^{c}**
Kaoma	165 (42.53)	65 (39.39)	100 (60.61)	
Shangombo	60 (15.46)	24 (40.00)	36 (60.00)	
**Type of Facility**				
Health Post	135 (34.79)	70 (51.85)	65 (48.15)	0.141^{c}
Rural Health Centre	200 (51.55)	96 (48.00)	104 (52.00)	
Urban Health Centre	53 (13.66)	19 (35.85)	34 (64.15)	
**Respondent Position**				
Environmental Health T/O	18 (4.64)	12 (66.67)	6 (33.33)	0.343^{c}

Community Health Assistant	25 (6.44)	10 (40.00)	15 (60.00)	
Community Health Worker	266 (68.56)	127 (47.74)	139 (52.26)	
Community Health Volunteer	79 (20.36)	36 (45.57)	43 (54.43)	
**Supervisor Position**				
Clinician	120 (30.93)	49 (40.83)	71 (59.17)	**<0.0001**[c]
Environmental Health T/O	172 (44.33)	101 (58.72)	71 (41.28)	
Other	96 (24.74)	35 (36.46)	61 (63.54)	
**Transport availability**				
Not available	279 (71.91)	108 (38.71)	171 (61.29)	**<0.0001**[c]
Rarely available	41 (10.57)	26 (63.41)	15 (36.59)	
Readily available	68 (17.53)	51 (75.00)	17 (25.00)	

c- Pearson chi^2 test, f= Fisher's exact test, wr= Wilcoxon rank-sum test

More than half of the participants, 58.91% (228/388), were knowledgeable about facial and environmental hygiene promotion and 52.06% (202/388) indicated that the intervention was relevant for trachoma control. More than half of the participants, 56.19% (218/388) were not trained in F and E. A third of the participants, 33.25% (129/388), had very positive attitude towards conducting F and E activities if MDA has recently been conducted in an area. Very few participants, 10.82% (42/388), had very negative attitude towards this. Most variables, perceived complexity, perceived relevance, training, incentives, availability of tools and IEC material and attitude were associated with adoption of F and E hygiene promotion, while knowledge was not (Table 1b).

Table 1b. Characteristics of the health care workers; Western Province, April 2023 (N=388)

**Factor**	**Frequency (%)**	Adopted F and E hygiene promotion	Did not adopt F and E	P-values

		n (%)	hygiene promotion n (%)	(Chi-square/Fisher's Exact/ Wilcoxon Rank sum)
**Knowledge on SAFE**				
Knowledgeable	228 (58.91)	110 (48.25)	118 (51.75)	0.741[c]
Not knowledgeable	159 (41.09)	74 (46.54)	85 (53.46)	
**Perceived complexity of F and E hygiene promotion**				
Not complex	252 (64.95)	136 (53.97)	116 (46.03)	**0.001[c]**
Complex	136 (35.05)	49 (36.03)	87 (63.97)	
**Perceived relevance of F and E hygiene promotion**				
Relevant	202 (52.06)	138 (68.32)	64 (31.68)	**<0.0001**
Not relevant	186 (47.94)	47 (25.41)	138 (74.58)	
**Training on F and E Promotion**				
Trained	170 (43.81)	94 (55.29)	76 (44.71)	**0.008[c]**
Not trained	218 (56.19)	91 (41.74)	127 (58.26)	
**Incentives**				
Provided	120 (30.93)	70 (58.33)	50 (41.67)	**0.005[c]**
Not provided	268 (69.07)	115 (42.91)	153 (57.09)	
**Tools and IEC material availability**				
Available	126 (32.47)	74 (58.73)	52 (41.27)	**0.003[c]**
Not available	262 (67.53)	111 (42.37)	151 (57.63)	
**Attitude- (It is important to include F and E hygiene messages in community outreach programs)**				
Very positive	238 (61.34)	119 (50.00)	119 (50.00)	**0.014[f]**

Positive	113 (29.12)	45 (39.82)	68 (60.18)	
Neutral	26 (6.70)	14 (53.85)	12 (46.15)	
Negative	5 (1.29)	1 (20.00)	4 (80.00)	
Very negative	6 (1.55)	6 (100)	0 (0.00)	
**Attitude- (There is need to conduct F and E activities if MDA has been conducted)**				
**Very positive**	129 (33.25)	69 (53.49)	60 (46.51)	**<0.0001**[c]
**Positive**	93 (23.97)	49 (52.69)	44 (47.31)	
**Neutral**	41 (10.57)	25 (60.98)	16 (39.02)	
**Negative**	83 (21.39)	20 (24.10)	63 (75.90)	
**Very negative**	42 (10.82)	22 (52.38)	20 (47.62)	

c- Pearson chi^2 test, f= Fisher's exact test, wr= Wilcoxon rank-sum test

Level of adoption of facial and environmental hygiene promotion

Out of the 388 participants, only 47.68% (185/388) of the respondents had adopted facial and environmental hygiene promotion. Of the three districts, Kalabo district had the highest proportion, 58.90% (96/163) of participants who had adopted facial and environmental hygiene promotion. Shangombo had the least proportion of respondents who adopted the intervention. See Fig 1.

Fig 1. Adoption of F and E hygiene promotion by district. The figure shows proportions of health workers who adopted the intervention by district. The orange portion of the bars represents the proportion of health workers who adopted the intervention whereas the purple portion represents the portion of health workers who did not adopt the intervention

The most widely adopted intervention strategy in the province was community leader engagement, with 65.46% (254/388) of health workers engaging community leaders when conducting

intervention activities. On the other hand, the least adopted strategy was community health education using IEC materials with only 24% (89/388) participating in it. Community leader engagement for promotion of environmental improvements and facial cleanliness was widely adopted in all the three districts, Shangombo 78.30% (47/60), Kaoma 64.20% (106/165) and Kalabo 62% (101/163) (Fig 2). Between the facial and environmental hygiene promotion core intervention components, community demonstrations for correct face and hand washing methods was widely adopted in Kaoma, 35.20% (58/165) and Kalabo, 52.10% (85/163).

Fig 2. Proportion participating in individual F and E hygiene promotion program components. The figure shows proportions adopting F and E program components in each district. The yellow bars represents the portion of health workers who participated in community leader engagement for F and E hygiene promotion, the dark blue bars represent those who conducted community demonstrations for face and handwashing methods, gray bars represent those who conducted F and E hygiene education in schools, orange bars represent those who participated in community distribution of IEC material and light blue bars those who attended stakeholder consultation meetings

Determinants of adoption of facial and environmental hygiene promotion

Results of univariate analysis showed that 10 variables were significantly associated with adoption, and 8 remained significant in the final multivariable logistic regression model (Table 2). Participants who were from Kalabo (AOR= 0.43, 95% CI = [0.24, 0.76]), and Shangombo (AOR = 0.38, 95% CI = [0.17, 0.83]) and those who were supervised by a profession other than an environmental health officer or a clinician (AOR = 0.47 (95% CI = [0.22, 1.03]) were significantly

less likely to adopt the intervention , On the other hand, participants who had readily available transport (AOR = 3.04. 95% CI = [1.38, 6.70]), who perceived the intervention as not being complicated (AOR = 2.47, 95% CI = [1.39, 4.40]), who perceived the intervention as relevant for trachoma control (AOR = 8.15, 95% CI = [8.15, 95% CI = [4.59, 14.46]), who had received training in facial cleanliness and environmental improvements (AOR = 2.19, 95% CI = [1.26, 3.79]), who had very positive attitude towards conducting facial and environmental hygiene promotion in communities where MDA has recently been conducted (AOR = 3.19, 95% CI = [1.41, 7.23]), who had neutral attitude towards conducting F and E hygiene promotion in communities where MDA has recently been conducted (AOR = 7.19, 95% CI = [2.65, 19.50]), and who had positive attitude towards conducting F and E hygiene promotion in communities where MDA had recently been conducted (AOR = 3.86, 95% CI = [1.61, 9.22]) were significantly more likely to adopt the intervention.

On the other hand, this study found that there was no significant association between the dependent variable adoption of the intervention and the independent variables, sex, length of service, level of education and respondent’s position.

Table 2. Binary and Multivariable Logistic Regression Results

**Explanatory Variable**	**Unadjusted Estimates**			**Adjusted Estimates**		
	OR	(95%CI)	P-value	AOR	(95%CI)	P-value
**Sex**						
**Female**	-	-	-			
Male	1.00	(0.67, 0.49)	0.989			
Age	1.002	(1.000, 1.003)	**0.042**			

**Service length**	1.02	(0.99, 1.05)	0.259			
**Education**						
No education	-	-	-			
Primary level	0.75	(0.14, 3.93)	0.733			
Secondary level	0.89	(0.18, 4.50)	0.887			
Tertiary level	1.77	(0.31, 10.09)	0.521			
**District**						
Kalabo	-	-	-	-	-	-
Kaoma	0.45	(0.29, 0.71)	**<0.0001**	0.43	(0.24, 0.76)	**0.004**
Shangombo	0.47	(0.25, 0.85	**0.013**	0.38	(0.17, 0.83)	**0.016**
**Respondent's position**						
Environmental Health Officer	-	-	-	-	-	-
Table 2 Continued						
Community Health Assistant	0.33	(0.09, 1.18)	0.089			
Community Health Worker	0.46	(0.17, 1.26)	0.129			
Community Health Volunteer	0.42	(0.14, 1.23)	0.113			
**Supervisor's position**						
Clinician	-	-	-	-	-	-
Environmental Health Officer	2.06	(1.28, 3.31)	**0.003**	1.56	(0.82, 2.98)	0.181
Other	0.83	(0.48, 1.45)	0.513	0.47	(0.22, 1.03)	0.060
**Facility transport type**						
Bicycle	-	-	-	-	-	-
Motor cycle	0.59	(0.28, 1.24)	0.165	0.49	(0.017, 1.36)	0.16

Motor vehicle	1.69	(0.50, 5.70)	0.397	3.86	(1.11, 13.59)	**0.033**
None	0.37	(0.22, 0.61)	**<0.0001**	0.68	(0.33, 1.39)	0.287
**Transport availability**						
Not available	-	-	-	-	-	-
Rarely available	2.74	(1.39, 5.42)	**0.004**	1.93	(0.73, 5.09)	0.183
Readily available	4.75	(2.61, 8.66)	**<0.0001**	3.04	(1.38, 6.70)	**0.006**
**Perceived intervention complexity**						
Complex	-	-	-	-	-	-
Not complex	2.08	(1.36, 3.20)	**0.001**	2.47	(1.39, 4.40)	**0.002**
**Training on F and E**						
Not trained	-	-	-	-	-	-
Trained	1.73	(1.15, 2.59)	**0.008**	2.19	(1.26, 3.79)	**0.005**
**Tools and IEC material**						
Not available	-	-	-	-	-	-
Available	1.94	(1.26, 2.98)	**0.003**	2.94	(1.65, 5.25)	**<0.0001**
**Attitude- (There is need to conduct F and E activities if MDA has recently been conducted)**						
Table 2 Continued						
Negative	-	-	-	-	-	-
Positive	3.51	(1.84. 6.71)	**<0.0001**	3.86	(1.61, 9.22)	**0.002**

Neutral	4.92	(2.20, 11.01)	**<0.0001**	7.19	(2.65, 19.50)	**<0.0001**
Very Negative	3.47	(0.88, 7.61)	0.402	4.15	(0.91, 12.19)	0.407
Very positive	3.62	(1.79, 6.67)	**<0.0001**	3.19	(1.41, 7.23)	**0.005**

Discussion

This study sought out to determine the level of adoption of facial and environmental hygiene promotion among health care workers and identify the determinants of adoption of this intervention. The study found that slightly less than half of the participants (47.68%) adopted the intervention and of the three districts included in the study, Kalabo had the highest proportion of participants who had adopted the intervention and Shangombo had the lowest proportion. The determinants of adoption of facial and environmental hygiene promotion included district of respondent, transport availability, perceived complexity of the intervention, perceived relevance of the intervention, training, availability of tools and IEC material and attitude towards the need to conduct facial and environmental hygiene promotion activities in communities that have recently undergone mass drug administration.

The low level of adoption may be inadequate to significantly contribute to intended population level outcomes in terms of reducing trachoma prevalence. According to Glasgow et al, for any intervention to be able to produce high impact on population health outcomes, all sites and individuals invited to participate must adopt the intervention [12]. However, this study found that less than half of the health workers invited to participate in the intervention adopted the intervention and this could possibly contribute to the slow gains towards trachoma elimination in the province. This assertion is supported by previous studies which showed that areas that highly adopt facial and environmental hygiene promotion achieve control faster [15, 16]. A study that

was conducted to review how the ‘SAFE’ strategy was implemented in Ghana, one of the first countries to achieve elimination, found that contrary to our finding, all the components of the ‘SAFE’ strategy, including facial and environmental hygiene promotion were implemented with equal and high level intensity [17, 18]. The level of adoption found in this study is consistent with that found in previous literature which similarly reported low adoption of the F and E components [8, 11]. The study by Asfaw et al (2020) found that only 44% and 24% of study settings adopted and implemented F and E hygiene promotion respectively. However, this study looked at adoption using implementation settings as the study units, so it is difficult to say whether our findings are comparable.

The most widely adopted strategy for facial and environmental hygiene promotion was community leader engagement for the promotion of facial and environmental hygiene in their communities as almost seventy percent of the respondents had participated in this. This finding was expected because engaging the community leaders (Indunas) in any programs taking place in the community is a cultural norm in western province of Zambia. Conducting demonstrations for correct face and handwashing methods in the communities was moderately adopted. These findings mean that F and E promotion is not being done according to what is recommended by WHO in Western Province of Zambia. The WHO and other trachoma stakeholders currently encourage countries implementing the ‘SAFE’ strategy to scale up the implementation of behavioral change strategies such as community demonstrations for correct face and handwashing methods [7, 19]. This study however finds that this is not the case in Western Province, Zambia as not many health workers adopted this strategy. Similarly, the review of grey literature on interventions to improve facial cleanliness & environmental improvement for trachoma prevention and control by [19] found that most of the adopted interventions for facial and environmental hygiene promotion were those that

focused on F and E-related health information dissemination, and very few interventions targeted behavior change activities. This could probably be due to a lack of research on proximal precedents of behavior such as attitudes and norms which makes it hard to design behavioral change interventions [19]. The study by Delea et al (2018) systematically reviewed many studies that were done in different settings, giving strong evidence that the observation we made in this study could be the case in many other 'SAFE' implementing settings.

Participants from Kaoma and Shangombo districts were less likely to adopt facial and environmental hygiene promotion compared to participants from Kalabo. This could have been because most of the participants with positive attitude towards the need to conduct F and E hygiene promotion in communities which have recently undergone MDA were from Kalabo compared to the other two districts. This is supported by previous studies by [9, 20] which found that attitude was an important determinant of adoption of F and E health promotion. Our finding regarding disproportionate adoption of 'SAFE' strategy interventions in different implementation sites of an endemic area are consistent with those of another study which evaluated trachoma control programs in eight countries and found that some endemic districts of Ghana and Mali did not widely adopt facial and environmental hygiene promotion interventions compared to other districts [21].

Transport availability for use in health promotion activities affected adoption of facial and environmental hygiene promotion. Majority of the participants indicated not having any available transport for use in community health promotion activities and these were less likely to adopt the intervention compared to those who indicated that such transport was rarely or readily available. This finding is not surprising as lack of transport makes it hard for health workers to carry out health promotion activities especially those involving community work. This finding is consistent

with those of previous studies which found that organizations implementing facial cleanliness and environmental improvement interventions did not receive enough funding to conduct sufficient activities in their districts compared to the organizations focusing on surgery and antibiotics interventions and mainly lacked transport [9, 10]. Our findings are similar to those of Tsang et al (2021) perhaps due to the fact that both research were conducted in rural areas and the study populations were very similar.

Both perceived complexity and perceived relevance of the intervention affected the adoption of facial and environmental hygiene promotion. Participants who found the intervention complicated and hard to implement were less likely to adopt it compared to those who felt it was uncomplicated and easy to implement. Similarly, participants who perceived the intervention as being relevant in trachoma prevention were more likely to adopt it compared to those who perceived it as not relevant in trachoma prevention. This may be because people who perceive an activity as complicated may not be motivated to engage in that activity and similarly people who do not perceive an activity as important may also not be motivated to engage in it. These finding are consistent with previous studies. A study in Nigeria found that respondents in schools had less knowledge on trachoma, its prevalence and the importance of facial cleanliness and environmental improvements in trachoma prevention and therefore had less confidence in teaching about hygiene practices and trachoma prevention [20]. Further, a systematic review by Maritim et al found that health worker perceptions of their ability to carry out health and sanitation education for trachoma control influenced their likelihood to participate in the activities [22].

IEC materials are a very important component of health promotion activities as they make it easy to deliver health education information to the community and may motivate staff to participate in health promotion activities. Our study found that participants who had IEC material for facial and

environmental hygiene promotion available were more likely to adopt the intervention compared to those who did not have any IEC material. This finding could have been so because IEC material informs health workers and provide a guide on what needs to be included in sensitization and as such make it easy for health workers to carry out health promotion activities. This gives health workers the motivation to carry out health promotion activities such as facial and environmental hygiene promotion. This finding is consistent with the previous study by [9] who similarly carried out their study in a rural and low resource area.

Previous studies have shown that training is important in trachoma health promotion activities [9, 20]. Our study found that participants who had received some training in facial cleanliness and environmental improvements components were more likely to adopt the intervention compared to those who had not received such training. This could be because participants who have been trained are more likely to be knowledgeable and confident to carry out the intervention activities compared to those who are not trained. This finding is consistent with previous studies such as [22] who found that easy access to information through educational materials and training for those involved in 'SAFE' implementation ensured proper service delivery.

A limitation of this study is that there was a potential for clustering of the quantitative data at district and facility levels which could affect the estimated standard errors and ultimately the generalizability of the study findings. To mitigate this, we reported robust estimates of odds ratios and confidence intervals at both binary and multivariable logistic regression to counter any possible clustering effects. This study found that Shangombo, a district which did not widely adopt community demonstration for correct face and hand washing methods did not record a reduction in trachoma prevalence. This may suggest that failure to implement such behavior change

~~interventions may lead to continued disease transmission~~, Future studies can test this hypothesis using experimental of quasi-experimental designs.

Conclusion

This study found that Adoption of facial and environmental hygiene promotion was low and the determinants of adoption of facial and environmental hygiene promotion included factors such as resource availability (transport, IEC materials and tools), positive attitude towards conducting F and E promotion if MDA is done, positive perception of intervention relevance and perception of non-complexity of intervention. There is need to increase funding towards facial and environmental hygiene promotion and provide training of health workers on facial and environmental hygiene promotion

Acknowledgements

Martha Kasongo is a recipient of a TDR scholarship under the Postgraduate Training Scheme in Implementation Research at the University of Zambia. We are grateful for the financial support for the training scheme provided by UNICEF, the UNDP, the World Bank and the WHO Special Program for Research and Training in Tropical Diseases (TDR)

References

1. Hu VH, Harding-Esch EM, Burton MJ, Bailey RL, Kadimpeul J, Mabey DCW. Epidemiology and control of trachoma: systematic review. Trop Med Int Health. 2010;15(6):673-91.
2. MoH. Elimination of Neglected Tropical Diseases National Masterplan. Government of the Republic of Zambia. 2019.

3. Tian L, Wang NL. Trachoma control: the SAFE strategy. International journal of ophthalmology. 2018;11(12):1887-8.
4. Stocks ME, Ogden S, Haddad D, Addiss DG, McGuire C, Freeman MC. Effect of water, sanitation, and hygiene on the prevention of trachoma: a systematic review and meta-analysis. PLoS Med. 2014;11(2):e1001605.
5. Paul Emerson and Laura Frost wRBaDM. Implementing the SAFE Strategy for Trachoma Control. International Trachoma Initiative. 2006.
6. MoH. Zambia "SAFE" Strategy Implementation Framework Directorate of Clinical Care and Diagnostic Services National Eye Health Coordination. 2013;1:41 - 4.
7. WHO. Informal consultation on end-game challenges for trachoma elimination, Task Force for Global Health, Decatur, United States of America, 7–9 December 2021. Geneva: World Health Organization; 2022 2022.
8. Asfaw M, Zolfo M, Negussu N, Tadesse F, Tadele T, Sisay A, et al. Towards the trachoma elimination target in the Southern region of Ethiopia: How well is the SAFE strategy being implemented? Journal of infection in developing countries. 2020;14(6.1):3s-9s.
9. Tsang K, de Wildt G, Mwingira U, Mtuy TB. Implementing trachoma control programmes in marginalised populations in Tanzania: A qualitative study exploring the experiences and perspectives of key stakeholders. PLOS Neglected Tropical Diseases. 2021;15(9):e0009727.
10. Wright HR, Keeffe JE, Taylor HR. Barriers to the Implementation of the SAFE Strategy to Combat Hyperendemic Trachoma in Australia. Ophthalmic Epidemiology. 2010;17(6):349-59.
11. ICTC. A call to action: Why ICTC is bringing the trachoma community together to advance facial cleanliness and environmental improvement. https://wwwtrachomacoalitionorg/news-

blogs/call-action-why-ictc-bringing-trachoma-community-together-advance-facial-cleanliness-and. 2023.

12. Glasgow RE, Harden SM, Gaglio B, Rabin B, Smith ML, Porter GC, et al. RE-AIM Planning and Evaluation Framework: Adapting to New Science and Practice With a 20-Year Review. Frontiers in Public Health. 2019;7.
13. Setia M. Methodology series module 3: Cross-sectional studies. Indian Journal of Dermatology. 2016;61(3):261-4.
14. Churko C, Asfaw MA, Zerdo Z. Knowledge, Attitude, Practices and Associated Factors Towards Trachoma Among People Living in Arba Minch Zuria District, Gamo Zone, Southern Ethiopia. Clin Ophthalmol. 2021;15:3075-85.
15. Roba A, Wondimu A, Patel D, Zondervan M. Effects of intervention with the SAFE strategy on trachoma across Ethiopia. Journal of Epidemiology & Community Health. 2011;65(7):626-31.
16. Pinsent A, Burton MJ, Gambhir M. Enhanced antibiotic distribution strategies and the potential impact of facial cleanliness and environmental improvements for the sustained control of trachoma: a modelling study. BMC Medicine. 2016;14(1):71.
17. Aboe A, Bush S, Faal H. The elimination of blinding trachoma in Ghana through improving access to water and latrines. 2011.
18. Yayemain D, King JD, Debrah O, Emerson PM, Aboe A, Ahorsu F, et al. Achieving trachoma control in Ghana after implementing the SAFE strategy. Transactions of The Royal Society of Tropical Medicine and Hygiene. 2009;103(10):993-1000.

19. Delea MG, Solomon H, Solomon AW, Freeman MC. Interventions to maximize facial cleanliness and achieve environmental improvement for trachoma elimination: A review of the grey literature. PLoS Negl Trop Dis. 2018;12(1):e0006178.
20. Lange FD, Jones K, Ritte R, Brown HE, Taylor HR. The impact of health promotion on trachoma knowledge, attitudes and practice (KAP) of staff in three work settings in remote Indigenous communities in the Northern Territory. PLoS Negl Trop Dis. 2017;11(5):e0005503.
21. Kuper H, Solomon AW, Buchan JC, Zondervan M, Mabey D, Foster A. Participatory evaluations of trachoma control programmes in eight countries. Trop Med Int Health. 2005;10(8):764-72.
22. Maritim P, Zulu JM, Jacobs C, Chola M, Chongwe G, Zyambo J, et al. Factors shaping the implementation of the SAFE strategy for trachoma using the Consolidated Framework for Implementation Research: a systematic review. Glob Health Action. 2019;12(1):1570646-.

Figure on Adoption of F and E hygiene promotion by district

Click here to access/download;Figure;Figure1.tif

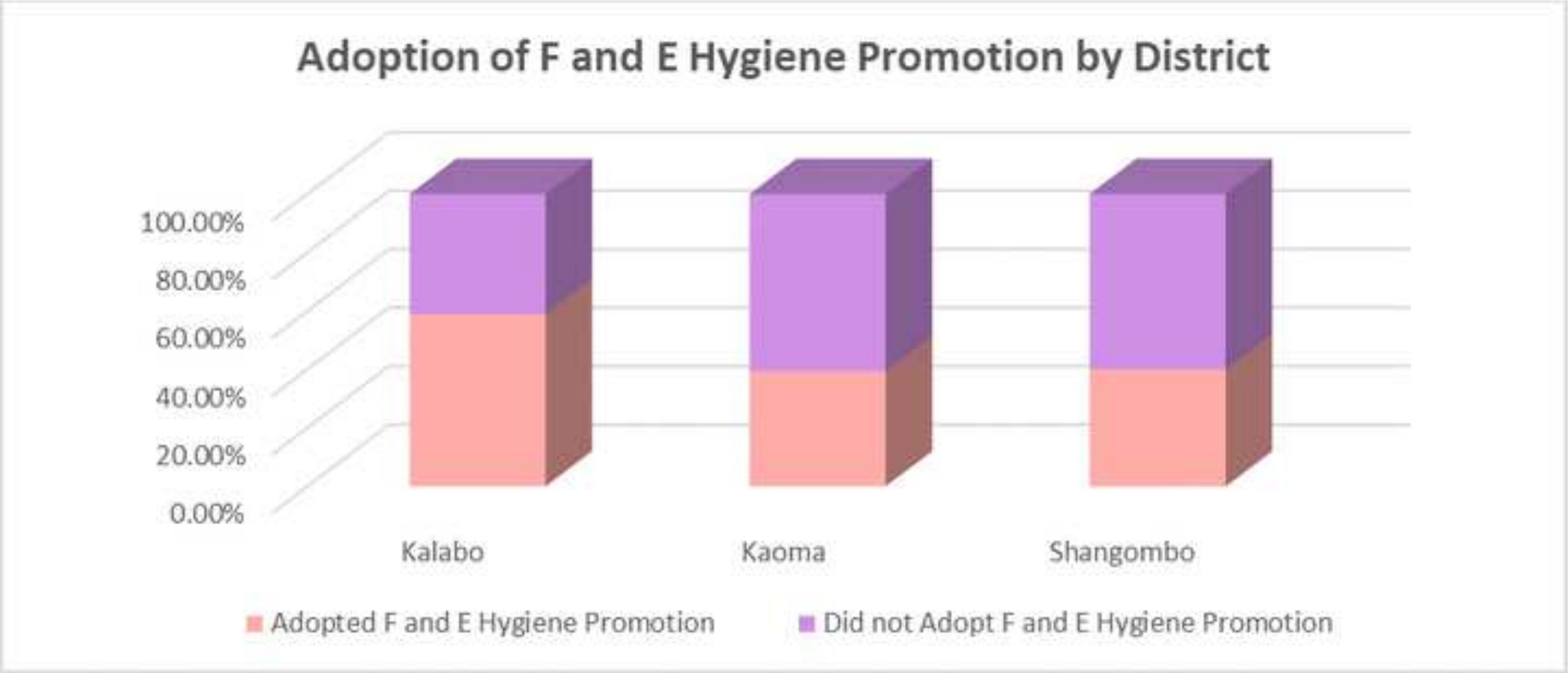

Figure on Proportion participating in individual F and E hygiene promotion program components

Click here to access/download;Figure;Figure2.tif

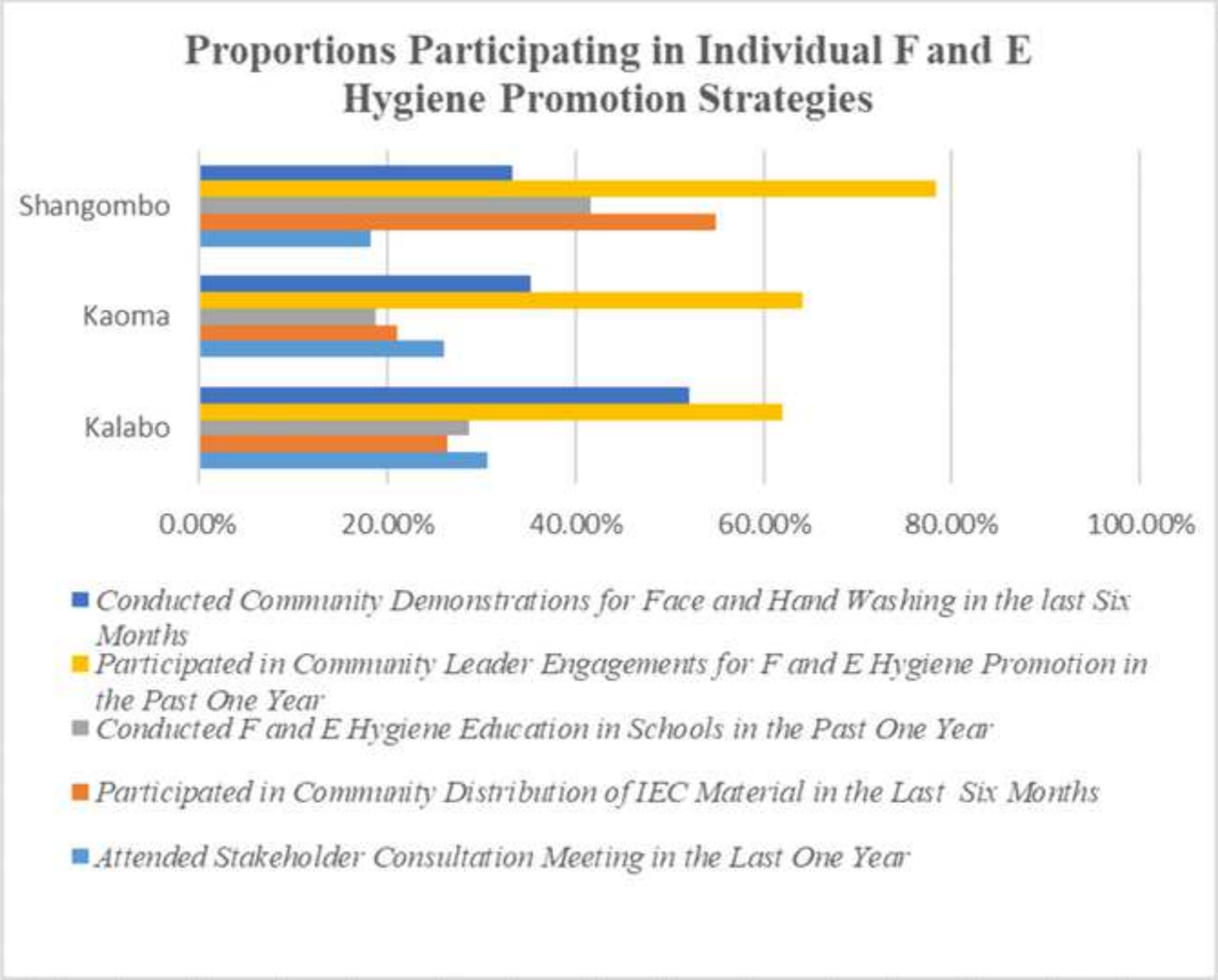

Data set

Click here to access/download
Supporting Information
Trachoma F and E.xls

Ethical Approval

Click here to access/download
Supporting Information
Ethical Approval.pdf
