# Supplementary material for: Factors associated with health worker adoption of facial and environmental hygiene promotion in the ‘SAFE strategy’ for trachoma elimination in Western Province, Zambia
Source: PLoS Negl Trop Dis. 2025 Sep 8;19(9):e0013483. doi: 10.1371/journal.pntd.0013483 (PMC12431646; doi:10.1371/journal.pntd.0013483)
Supplement: Attachment — Submitted filename: renamed_e746a.docx [file pntd.0013483.s003.docx]

Under key words – consider rephrasing to Determinants; Adoption; Facial and environmental hygiene promotion; SAFE strategy; Trachoma elimination; Western Province, Zambia as key words

Line 22 – consider including this statement; In Zambia, Western province is the most affected province with trachoma. Ref this in the main body^1-3^Reference this work to justify selection of Western province for this research

1.	Mwale C, Mboni C, Saasa N, et al.; Assessing trachoma elimination progress in districts with persistent trachoma, Western Province, Zambia. *International health* 2025:ihaf041.

2.	Mwale C, Mumbi W, Funjika M, et al.; Prevalence of trachoma in 47 administrative districts of Zambia: results of 32 population-based prevalence surveys. *Ophthalmic epidemiology* 2018;**25**(sup1):171-180.

3.	Mwale C, Mulamba M, Chama E, et al.; Assessment of outcomes of the patients undergoing surgery for Trachomatous Trichiasis, Western Province, Zambia. *Medical Journal of Zambia* 2025;**52**(3):332-339.

Line 1 – edit to read Determinants of adoption of facial and environmental hygiene promotion in the ‘SAFE strategy’ for trachoma elimination in Western Province, Zambia – a cross-sectional study or

Determinants of adoption of facial and environmental hygiene promotion in the ‘SAFE strategy’ for trachoma elimination in Western Province, Zambia

Line 22 – Rephrase to The SAFE strategy (Surgery, Antibiotics, Facial Cleanliness 23 and Environmental Improvement) is recommended by WHO for elimination of trachoma.

Line 52 – elimination of trachoma in endemic communities

Line 63 – elimination program officers and stakeholders

Ine 72, include references 1 and 2 above

Line 102 - help trachoma elimination program implementers identify possible solutions to enhance adoption

Line 152 - equation, n ≥ (Z2 p [1-p])/e2 where, n is the sample size, z is the selected critical value of desired – state the value of each parameter used in the equation

Line 147 - Sampling – where facility in charges included among participants, if not why?

Line 389 - This study found that Shangombo, a district which did not widely adopt community demonstration for correct face and hand washing methods did not record a significant reduction in trachoma prevalence. – ref 1 above

Line 392 interventions may lead to persistent active trachoma – ref 1 above

Line 398 – what is MDA
