# Supplementary material for: Factors associated with health worker adoption of facial and environmental hygiene promotion in the ‘SAFE strategy’ for trachoma elimination in Western Province, Zambia
Source: PLoS Negl Trop Dis. 2025 Sep 8;19(9):e0013483. doi: 10.1371/journal.pntd.0013483 (PMC12431646; doi:10.1371/journal.pntd.0013483)
Supplement: Attachment — Submitted filename: Responses to Reviewers.docx [file pntd.0013483.s005.docx]

Martha Kasongo,

Department of Epidemiology and Bistatistics,

School of Public Health, University of Zambia, Zambia

6^th^ August 2025

The Editor,

PLOS Neglected Tropical Diseases

**RE: Resubmission of revised manuscript for publication in your journal**

We would like to thank the reviewers for taking their time to review our manuscript and for their valuable comments.

We wish to resubmit a revised version of manuscript for your consideration. The title of the manuscript is “**Factors associated with health worker adoption of facial and environmental hygiene promotion in the ‘SAFE strategy’ for trachoma elimination in Western Province, Zambia.**”

A Point-by point responses (in blue) to the comments raised by reviewers are shown below. In addition, one ‘clean’ and one 'tracked' version of our revised manuscript have been uploaded; the marked version indicating the changes made to the manuscript during revision.

Sincerely,

Martha Kasongo

Email: [mkasongo10@gmail.com](mailto:mkasongo10@gmail.com)

**Responses to reviewers’ comments**

**REVIEWER #1**

**Methods**

1. In Background (page 2), the authors didn't explain about the reason of the "interventions particularly for facial cleanliness and environmental improvement have been sub-optimally adopted"

Thank you. We have now added an explanation of the possible reasons of the sub-optimal adoption of the intervention by adding the sentence “due to lack of prioritization and inadequate funding of intervention activities” in the background. Page 2, line 25

2. In Introduction (page 6), the authors have to explain, in depth, the usefulness of the results. The authors only mentioned: "The results will inform......through the identified determinants of adoption"

Thank you for this guidance. A detailed explanation on the usefulness of the results has now been included. However, this information has been put under the conclusion. Page 23 - 24, line 415 - 428

3. In Methods (page 6), the study employed the RE-AIM/PRISM but it's not clear if there some evidence of the use of RE-AIM/PRISM in trachoma's studies

Thank you. This section has been revised and a paragraph discussing usage of the RE-AIM/PRISM framework in previous studies has been included. Further, examples of some of these previous studies have been cited in the paragraph “Although the RE-AIM framework has been used in previous trachoma studies, many have largely concentrated on assessing reach and effectiveness of trachoma interventions [1, 2]. No studies have attempted to assess and identify the contextual factors shaping adoption of trachoma control interventions among health providers. A systematic review of interventions used the RE-AIM framework to identify effective and ineffective strategies for addressing barriers to the uptake of eye care services in developing countries [1]. Another study in Niger used the RE-AIM framework to assess reach of azithromycin distribution for child survival by comparing door to door and fixed delivery strategies [2]”. Page 6 - 7, line 127 – 134.

4. The authors also mentioned the following: "it is one of the understudied areas in this field". I recommend to explain the idea in introduction

Thank you. The section has now been revised and the statement “Like other low income countries, little is known about the factors associated with adoption of facial and environmental hygiene promotion in Zambia as no study has looked into adoption of the F and E components of the SAFE strategy in the country. Few studies conducted in other regions have assessed implementation of the SAFE strategy interventions but it is unclear if their findings are generalizable to Zambia [3, 4]” has now been included to highlight the research gap. Page 6, line 110 – 114.

5. In sampling and participant recruitment (page 8) stratified random sampling method was used so it's necessary to estimate the sample size calculation with design effect

Thank you so much for this important observation. We do agree that it is necessary to estimate the sample size calculation with design effect. However, we have not made any changes to the sample size. This is because we were unable to estimate the design effect of our sampling design due to limited availability of similar previous surveys and a lack of a pilot study from which to estimate variances. However, we believe that the sample of the study may still be sufficient because the design effect (deff) for stratified random sampling if done by incorporating proper stratification and random sampling within strata. [5]

6. I suggest to explain a little more some variables like training, position held, length of service, etc before the results. E.g. perceived intervention complexity, how it complexity define?
   e.g.2. transport availability, and the options are not available, rarely available and readily available. what does mean rarely? once, twice a month?

This has been worked on. We have included an explanation of the variables training, perceived relevance, perceived complexity, transport availability and attitude under a newly added section of the methodology, measurement of variables. The included explanation reads “Training was a dichotomous variable coded as yes if a participant had received training on F and E components of the SAFE strategy. IEC material availability was dichotomized, denoting presence or absence of IEC materials containing information on the ‘F’ and ‘E’ components of the SAFE strategy. This could be any type of IEC material that an individual health center might have made or obtained from other trachoma stakeholders or through any other means. Transport was classified as a categorical variable, categorized as readily available if it was accessible at least once per week, rarely available if accessible at least once per month but not weekly, and not available if it was either entirely absent or unavailable for a month or more. Perceived complexity of the intervention was measured as a dichotomous variable, based on whether respondents found facial and environmental hygiene promotion activities complicated and difficult to carry out. Similarly, perceived relevance was a dichotomous variable, determined by whether respondents believed that facial and environmental hygiene promotion was important for preventing trachoma transmission

Lastly, attitude towards facial and environmental hygiene promotion was assessed by asking participants how they felt about the statement: “There is no need for me to conduct facial and environmental hygiene promotion activities in a community where MDA has recently been conducted.” Response options included: strongly agree, agree, neutral, disagree, and strongly disagree, which were considered as reflecting attitudes ranging from very negative to very positive.” Page 10, line 195 - 212

7. In data collection (page 9), the authors mentioned that the quantitative data was collected by the principal investigator with help of 3 research assistants. I suggest to introduce more information about the research assistants, e.g. they trained before the study's execution

Some information about the research assistants has been introduced by adding the statement “The principal investigator collected the data with the help of 3 research assistants who were natives of the three districts included in the study. These research assistants received training on the study and data collection tool a week prior to the commencement of data collection.” Page 9, line 179 – 182.

**Results**

8. I don't understand why the authors show the characteristics of the participants in two tables. Is there some reason?

Yes, there was a reason for this. Initially all the characteristics of the participants were presented in one table and the table proved to be too long. We therefore split it into two. However, given that we have now removed a number of variables from the study including education, length of service, respondent’s position and knowledge, we have now merged Table 1a and Table 1b into one Table 1of characteristics of the participants. Page 12 - 14, line 256 – 258.

9. I recommend to write the main results (page 10). e.g. 34.79% were from health posts. I will change this instead 51.55 were from rural health centre

Thank you. This mistake in reporting of results was noted. It has now been corrected as advised and the statement now reads “A majority 51.55% (200/388) were from Rural Health Centers” Page 12, line 247 – 248.

**Conclusions**

10. The discussion is too long and it's not clear about the information that the authors want to give to the readers. e.g. Our findings are similar to those of Tsang et al (2021) perhaps due to the fact that both research were conducted in rural areas and the study populations were very similar (page 22).

The majority of the studies about trachoma were developed in rural setting so the information gave for the authors is not relevant

Thank you for this important observation. Indeed, we realized that the discussion was too long with some unclear parts. We have made corrections according to your recommendations. Some irrelevant information that seemed unclear have been removed and only very important findings have been discussed. Page 18- 23, lines 309 – 410.

**Editorial and Data Presentation Modifications**

11. I suggest only use the variables that are really important to the study. I think there are so many variables that it could create a confusion to the reader

Thank you so much for this important comment. We have now removed some variables such as education, length of service, respondent’s position and knowledge from the study. The current fewer variables have even made it possible to merge Table 1a and Table 1b into one (Table 1) of characteristics of the participants. Page 12 - 14, line 256 – 258.

**Summary and General Comments**

12. I will review the regression analysis carefully, as the authors used stratified random sampling. This sampling design requires adjustments in the regression model to avoid underestimating the confidence intervals. Maybe, Incorporate strata and cluster variables into the model.

Thank you for this very helpful guidance. We have now reviewed the regression analysis and have now incorporated both the cluster variable (district) and the strata variable (type of heath facility) into the model to avoid underestimating the confidence intervals. The estimates have only slightly changed. Page 16 - 18, line 303 - 306

**REVIEWER #2**

**Methods**

1. The main issue is that the sampling strategy was confusing. Is this a random sample?

We acknowledge that the sampling strategy as was presented was confusing. We have rectified this by adding more information on how the sampling was done at all the different levels. The added information reads “…Using probabilities proportional to size of the number of health workers in each district, the random sample sizes for each district were calculated as 166, 60 and 164 for Kaoma, Shangombo and Kalabo, respectively.

A total of 24 health facilities were randomly selected from a pool of 52 using probability proportional to size (PPS). Within each district, facilities were stratified by type including urban health centers, rural health centers, and health posts, and listed accordingly. A PPS-based random sample was then drawn from each stratum. Similarly, the number of health workers sampled from each facility was estimated using PPS based on the total number of health workers at each facility. Finally, systematic sampling was used to select participants at the health facility using a sampling frame provided by the facility in-charge. This was done by selecting every n^th^ person, calculated by dividing the total number of health workers listed on the sampling frame by the required sample size at a particular health facility. In a case were a participant did not give consent or was unreachable, the next name on the list was selected. Health workers were eligible to participate in the study if they were involved in community health promotion activities and had worked in their position for not less than one year.” Page 8, line 161- 175

**Conclusions**

2. Generally yes though in the limitations paragraph I would comment that these results may not be generalizable outside these districts of Zambia, and I would limit any conclusions only to the study area and not try to extrapolate to other settings.

This has been rectified and the statement “This study had two limitations. Firstly, using a stratified random sampling design potentially introduced clustering of the data at the different levels of the cluster and strata variables, signifying that our findings may not be generalizable outside the three districts of Zambia…” has been included in the limitation paragraph. Page 22, line 402 – 404.

**Summary and General Comments**

3. Line 33: what intervention? It’s not clear what these percentages refer to

Thank you for pointing this out. We have corrected this by adding a sentence to explain how adoption was defined in our study. This sentence has been added in the methodology section of the abstract and it reads “Adoption of facial and environmental hygiene promotion was self-reported and defined as having participated in community distribution of information, education and communication (IEC) materials or community demonstrations of correct hand and face washing methods or both in the past six months” Page 2, line 33- 36

4. Line 34: not sure the lower odds in Kaoma and Shangombo need to be in the abstract. That seems like knowledge that would be important for local policymakers but this paper is presumably being written to share more broadly generalizable knowledge. I would delete that sentence. (And probably the final sentence of the conclusion)

Thank you. This was well noted and we have now deleted the information regarding the odds of adoption in Kaoma and Shangombo from the abstract

5. Line 40: similar comment, but need to specify what adoption is, and how it is defined. Is this self-reported adoption? What components were adopted?

The information about how adoption was defined in our study has been added to the methodology section of the abstract. The sentence included reads “Adoption of facial and environmental hygiene promotion was self-reported and defined as having participated in community distribution of information, education and communication (IEC) materials or community demonstrations of correct hand and face washing methods or both in the past six months” Page 2, line 33- 36.

6. Line 80: “core components”: is this promoted by the government in some official way? Is this a more informal assessment by the authors based on activities of NGOs? Please more clearly state how these core components have been defined.

Thank you. Yes, this is promoted by government. The Ministry of Health started implementing the full SAFE strategy in 2013 and to guide intervention activities, the Zambia “SAFE” Strategy Implementation Framework was developed and approved. This framework identifies community sensitizations through distribution of IEC materials and community demonstrations for correct face and handwashing methods as the key intervention activities for facial and environmental hygiene promotion. This information has now been added to the introduction by including the statement “In 2013, Zambia through the MoH and supporting non-governmental organizations such as Site Savers started implementing the full SAFE strategy for trachoma elimination in its endemic districts. To facilitate the implementation process, the MoH developed the Zambia SAFE Strategy Implementation Framework which provided guidelines on intervention activities. In Zambia, facial and environmental hygiene promotion is done by health workers in collaboration with community health workers to achieve community level adoption of facial cleanliness and environmental hygiene practices [6]. According to the Zambia SAFE Strategy Implementation Framework, the core components of facial and environmental hygiene promotion include community sensitization using information, education and communication (IEC) material and demonstrations for correct face and hand washing methods [6]. Other activities include stakeholder consultation meetings, promotion of these interventions in schools and engagement of community leaders to champion change in their communities [6].” Page 5 - 6, line 98 – 109

7. Line 81: Are IEC materials a standardized set of materials made by the government? Or by an NGO? Or does this just mean any type of materials that an individual health center might have made.

Thank you. In this study, IEC material meant any type of material that an individual health center might have made or obtained from other trachoma stakeholders or through any other means. This was defined as IEC material containing information on trachoma prevention through facial cleanliness and/or environmental improvements. To address your comment, we have now added a clear definition of what was meant by IEC material in the Methodology section under the measurement of variables sub-heading. The statement included reads …” IEC material availability was dichotomized, denoting presence or absence of IEC materials containing information on the ‘F’ and ‘E’ components of the SAFE strategy. This could be any type of IEC material that an individual health center might have made or obtained from other trachoma stakeholders or through any other means.” Page 10, line 196 – 199.

8. Line 136: “full SAFE strategy”: who makes this decision / who implements this. Is this from the government/MOH? Is this NGOs?

Thank you so much for this comment. To make this clearer, we have added a statement regarding who is responsible for implementing the “full SAFE strategy” under the introduction. The statement included reads ….”In 2013, Zambia through the MoH and supporting non-governmental organizations such as Site Savers started implementing the full SAFE strategy for trachoma elimination in its endemic districts. To facilitate the implementation process, the MoH developed the Zambia SAFE Strategy Implementation Framework which provided guidelines on intervention activities.” Page 5, line 98 – 101.

9. Line 156: the methods for stratified random sampling were not clear. In line 160, does “representative” = “random” (and if so, please use the word random). I wondered if 24 health facilities were randomly selected from the total pool of 52 health facilities using probability proportional to size sampling, and then if within these 24 selected facilities systematic sampling was employed. (but no details are given about how the systematic sampling was actually done, and my summary above is not actually stated anywhere but just my hypothesis of what was done.) Please be more explicit about the sampling.

Thank you so much for this comment. We have replaced the word “representative” with the word “random” as guided. Further, we acknowledge that the sampling strategy as was presented was confusing. We have rectified this by adding more information on how the sampling was done at all the different levels. The added information reads “..Using probabilities proportional to size of the number of health workers in each district, the random sample sizes for each district were calculated as 166, 60 and 164 for Kaoma, Shangombo and Kalabo, respectively.

A total of 24 health facilities were randomly selected from a pool of 52 using probability proportional to size (PPS). Within each district, facilities were stratified by type including urban health centers, rural health centers, and health posts, and listed accordingly. A PPS-based random sample was then drawn from each stratum. Similarly, the number of health workers sampled from each facility was estimated using PPS based on the total number of health workers at each facility. Finally, systematic sampling was used to select participants at the health facility using a sampling frame provided by the facility in-charge. This was done by selecting every n^th^ person, calculated by dividing the total number of health workers listed on the sampling frame by the required sample size at a particular health facility. In a case were a participant did not give consent or was unreachable, the next name on the list was selected. Health workers were eligible to participate in the study if they were involved in community health promotion activities and had worked in their position for not less than one year.” Page 8, line 161- 175.

10. Line 222: how was “knowledgeable about facial and environmental hygiene promotion” defined? Is this knowledge? Or familiarity?

To assess knowledge in this study, a total of five multiple choice questions on trachoma and facial cleanliness and environmental improvement components of the SAFE strategy were asked. The questions included, what is trachoma? What do the acronyms “SAFE” stand for? Which components of the SAFE are used for trachoma transmission prevention? What is the best approach to implementing the SAFE strategy? And what is facial and environmental hygiene promotion? The variable knowledge was then coded on a binary scale measured as knowledgeable if the respondent scored three or more correct responses out of five and as not knowledgeable if the respondent scored less than three correct responses out of the five questions. However, kindly note that this variable has been dropped from the analysis and results of its analysis removed from the manuscript. This was done as advised by reviewers to reduce the number of variables as too many would be confusing to the reader.

11. Line 225: how was a “positive attitude” defined, or how was the question asked? (If as stated in Table 1b, I would include this in the text as well)

Thank you for this important observation. We have now included an explanation on how attitude was assessed in this study. The statement reads “…attitude towards facial and environmental hygiene promotion was assessed by asking participants how they felt about the statement: “There is no need for me to conduct facial and environmental hygiene promotion activities in a community where MDA has recently been conducted.” Response options included: strongly agree, agree, neutral, disagree, and strongly disagree, which were considered as reflecting attitudes ranging from very negative to very positive.” Page 10, line 207 – 212.

12. Table 1b: “perceived relevance of F and E”: relevance for what? Relevant for trachoma control? Relevant as an activity that the health center should be doing?

Thank you for this important comment. This was perceived relevance of facial and environmental hygiene promotion in prevention of trachoma transmission in communities. In order to make this clear, we have included an explanation of what was meant by including the statement “…Similarly, perceived relevance was a dichotomous variable, determined by whether respondents believed that facial and environmental hygiene promotion was important for preventing trachoma transmission.” Under measurement of variables in the methods. Page 10, line 204 - 206

13. Table 1b: what does “incentives” mean. That the workers are paid extra to do hygiene promotion above and beyond their salary?

Yes, incentives meant that the workers were given a token of appreciation for conducting health promotion activities for facial and environmental hygiene. This could be in monetary form or other, above and beyond their salary. However, kindly note that this variable has been dropped from the analysis and results of its analysis removed from the manuscript. This was done as advised by reviewers to reduce the number of variables as too many would be confusing to the reader.

14. Table 1c: spell out full words of IEC in footnote or caption

This has now been done Page 14, line 258 – 259.

15. Table 2: I believe the AOR was constructed from all variables in the table after applying a backwards stepwise approach.
(A) what was the p-value threshold to remove a variable from the model? (maybe include this in the methods)

Thank you. The p-value threshold to remove a variable from the model was p < 0.05. This information has now been included in the methods under the data analysis section. The statement now included reads “…Variables were declared statistically significant at p < 0.05” Page 11, line 231 – 233.

(B) in the table caption or footnote I would include that the adjusted model was selected using a backwards stepwise approach.

This information has been included in the notes about the table written just below the table Page 18, line 305 - 306.

(C) in the table caption please specify what the outcome is for the ORs (adoption of promotion activities I believe?)

This has now been specified in the table caption Page 16, line 303

Thank you so much for highlighting these omissions.

16. Line 288: “determinants” makes this sound like a causal relationship. But here only an association has been found. I would change to “The main factors associated with…” or something like that. Also: I did not scour the text after noticing this, but please read through and make sure that the text uses words that expresses only a cross-sectional association, and not a causal relationship.

Thank you for this important observation. We have now replaced the word “determinants) with the phrase “factors associated with..” wherever it was applied.

17. Line 391: trachoma prevalence was not reported here. A citation should be given, and also a little more detail. (What does a “decrease in trachoma prevalence” mean? Between which years? According to which surveys? Does this mean TF?)

We have now replaced the information which was earlier presented as it was indeed unclear and referred to prevalence from earlier surveys. We have now replaced this information with information from the most recent surveys and a citation has been given. The information now included reads “…a study assessing trachoma elimination progress in districts with persistent trachoma in Western Province, Zambia, reported that the prevalence of TF in children aged 1–9 years only declined from 17.9% to 12.5% between 2018 and 2023 in Shangombo. This indicates that trachoma remains a public health problem in the district, meeting the WHO criteria for persistent trachoma [7].” Page 22, line 389 – 393.

18. Line 299: “there is a need to increase funding”: I am not sure I agree. If I were a donor I would want evidence that implementing F&E is effective. Currently there are very few interventional studies that have shown F&E to be effective. Almost all the evidence comes from observational studies. If health workers are not adopting hygiene promotion but hygiene promotion does not work, then why should funders devote more money to it? Also, the results of this one study may not be relevant to other places. You could consider some caveats to this sentence, eg: “The results of this study demonstrate a need for increased funding and training in facial and environmental hygiene promotion in the study area, given the district/zone/federal stated plans/commitment/goals for hygiene programming.” Or something like that, which would be accurate for the Zambian setting.

Thank you for this guidance. The conclusion has now been revised and the statement you have highlighted has been rephrased to now read “Health workers with easy access to transport and IEC materials were more likely to adopt the intervention, highlighting the need for consistent resource availability. This can be addressed through increased funding and budget allocation for facial and environmental hygiene promotion activities, especially given the Government of Zambia’s commitment to implementing all components of the SAFE strategy.” Page 23, line 415 - 420

19. Fig 1: these are petty comments, and if it is very difficult to change I would not worry about it, but for your next paper consider: (a) no need for any decimal places on y axis; (b) third dimension on bar graphs conveying no information and thus should be dropped.

Thank you so much for this guidance. We have removed the decimal places from the y-axis.

**REVIEWER #3**

**Methods**

1. The objectives of the study are clearly articulated with a clear testable hypothesis stated

The study design is appropriate to address the study objectives

The targeted and sampled population is clearly described and appropriate for the hypothesis being tested

The sample size is sufficient to ensure adequate power to address the hypothesis being tested

The study applied the correct statistical analysis to support conclusions

The study has no concerns about ethical or regulatory requirements. All ethical or regulatory requirements were met

Thank you so much

**Results**

2. The analysis which was conducted matches the analysis plan

The study results are clearly and fully presented

The figures (Tables, Images) included in the study are sufficient quality for clarity

Thank you so much

**Conclusions**

3. The conclusions are supported by the data presented, though the conclusion should be strengthened and rephrased to focus on key information from the results presented

Thank you. The conclusion has now been strengthened and rephrased to focus on key information from the results presented. The conclusion now reads “Overall, health worker adoption of facial and environmental hygiene promotion was low. Key factors influencing adoption included the availability of transport and IEC materials, attitudes toward facial and environmental hygiene promotion, and perceptions of the intervention’s relevance and complexity. Health workers with easy access to transport and IEC materials were more likely to adopt the intervention, highlighting the need for consistent resource availability. This can be addressed through increased funding and budget allocation for facial and environmental hygiene promotion activities, especially given the Government of Zambia’s commitment to implementing all components of the SAFE strategy. Additionally, health workers who had received training in F and E promotion were more likely to adopt the intervention than those who had not, underscoring the importance of targeted capacity building. Training should emphasize the relevance of facial and environmental hygiene promotion in trachoma prevention, provide clear guidance on how to implement the intervention, and address perceptions of complexity. Importantly, training should also reinforce the need to continue facial and environmental hygiene promotion even in communities where mass drug administration of antibiotics for trachoma has already been conducted. These insights should inform strategies to improve adoption of facial and environmental hygiene promotion, ultimately supporting population-level behavior change and advancing progress toward trachoma elimination.” Page 23, line 412 – 428.

4. The limitations of analysis should be clearly described

Thank you for this comment. Indeed, we realized that the limitations as earlier written were not clearly described. We have rephrased the paragraph on the earlier mentioned limitations to make it more clearly. Further, we have added another limitations which was omitted in the earlier manuscript regarding the potential for recall bias. The statement on limitations now reads “This study had two limitations. Firstly, using a stratified random sampling design potentially introduced clustering of the data at the different levels of the cluster and strata variables, signifying that our findings may not be generalizable outside the three districts of Zambia. To mitigate this, we report robust estimates of odds ratios and confidence intervals to counter clustering effects. Further, we incorporated the strata and cluster variables into the model to avoid underestimating the confidence intervals. Secondly, the use of a questionnaire to gather self-reported information on adoption of facial and environmental hygiene promotion is subject to recall bias. Participants could possibly recall wrongly that they had taken part in intervention activities when in fact they had not” Page 22 - 23, line 402 – 410.

**Summary and General Comments**

5. Under key words – consider rephrasing to Determinants; Adoption; Facial and environmental hygiene promotion; SAFE strategy; Trachoma elimination; Western Province, Zambia as key words

Thank you for this guidance. We have now rephrased the key words accordingly. Page 3, line 50 - 51

6. Line 22 – consider including this statement; In Zambia, Western province is the most affected province with trachoma. Ref this in the main body1-3Reference this work to justify selection of Western province for this research

Thank you. The statement “In Zambia, Western province is the most affected province with trachoma” has now been included in the abstract. Further, we have included this information in the main body and referenced the work you have recommended. The information included under the study setting reads “At the time of the study, Western Province had the highest prevalence of trachoma with some districts having as high as 17% prevalence of TF in children aged 1-9 years old [8, 9].” Page 7, line 141 – 143.

7. Line 1 – edit to read Determinants of adoption of facial and environmental hygiene promotion in the ‘SAFE strategy’ for trachoma elimination in Western Province, Zambia – a cross-sectional study or Determinants of adoption of facial and environmental hygiene promotion in the ‘SAFE strategy’ for trachoma elimination in Western Province, Zambia

Thank you for this guidance. We have edited the title as you have advised. Pease note that we were also advised by reviewers to change the word “determinants” to “factors.” The edited title therefore now reads “Factors associated with health worker adoption of facial and environmental hygiene promotion in the ‘SAFE strategy’ for trachoma elimination in Western Province, Zambia” Page 1, line 1 - 3

8. Line 22 – Rephrase to The SAFE strategy (Surgery, Antibiotics, Facial Cleanliness 23 and Environmental Improvement) is recommended by WHO for elimination of trachoma

Thank you. We have made this change. Page 2, line 23

9. Line 52 – elimination of trachoma in endemic communities

Thank you. We have made this change. Page 3, line 56

10. Line 63 – elimination program officers and stakeholders

Thank you. We have made this change. Page 4, line 66

11. Line 72, include references 1 and 2 above

Thank you for this guidance. We have included the mentioned references. Page 5, line 93 - 97

12. Line 102 - help trachoma elimination program implementers identify possible solutions to enhance adoption

Thank you. We have made this change. Page 6, line 115

13. Line 152 - equation, n ≥ (Z2 p [1-p])/e2 where, n is the sample size, z is the selected critical value of desired – state the value of each parameter used in the equation

Thank you. The values of each of the parameters used in the equation have now been stated. Page 8, line 155 - 160

14. Line 147 - Sampling – where facility in charges included among participants, if not why

Thank you. No, facility in charges were not included in the study because the study included only health workers involved in community health promotion activities. In the study setting, most facility in charges are nurses and clinical officers whose duties are facility based. Facility in charges would have only been included in a special case if the environmental health officer was also in charge of the facility. However, we did not encounter such a case in this study. To make this clear, we have now included this information on the eligibility criteria under the sampling and participant recruitment section in the methods. The included sentence reads “Health workers were eligible to participate in the study if they were involved in community health promotion activities and had worked in their position for not less than one year.” Page 8 - 9, line 173 – 175.

15. Line 389 - This study found that Shangombo, a district which did not widely adopt community demonstration for correct face and hand washing methods did not record a significant reduction in trachoma prevalence. – ref 1 above

Thank you. This sentence has been rephrased and the mentioned reference included. Page 22, line 389 – 393.

16. Line 392 interventions may lead to persistent active trachoma – ref 1 above

Thank you. This sentence has now been corrected according and the reference included. Page 22, line 392 – 393.

17. Line 398 – what is MDA, which appears in the conclusion

Thank you. It is mass drug administration of antibiotics and has now been written in full. Page 23 line 425 - 426.

REFERENCES

1.	Abdullah KN, Al-Sharqi OZ, Abdullah MT. Barriers to the uptake of eye care services in developing countries: a systematic review of interventions. Health Education Journal. 2013;72(6):742-54.

2.	Arzika AM, Maliki R, Amza A, Karamba A, Gallo N, Aichatou B, et al. Comparison of door-to-door and fixed-point delivery of azithromycin distribution for child survival in Niger: A cluster-randomized trial. PLOS Global Public Health. 2023;3(11):e0002559.

3.	Tsang K, de Wildt G, Mwingira U, Mtuy TB. Implementing trachoma control programmes in marginalised populations in Tanzania: A qualitative study exploring the experiences and perspectives of key stakeholders. PLOS Neglected Tropical Diseases. 2021;15(9):e0009727.

4.	Asfaw M, Zolfo M, Negussu N, Tadesse F, Tadele T, Sisay A, et al. Towards the trachoma elimination target in the Southern region of Ethiopia: How well is the SAFE strategy being implemented? Journal of infection in developing countries. 2020;14(6.1):3s-9s.

5.	Cochran WG. Sampling techniques: john wiley & sons; 1977.

6.	MoH. Zambia “SAFE” Strategy Implementation Framework Directorate of Clinical Care and Diagnostic Services National Eye Health Coordination. 2013;1:41 - 4.

7.	Mwale C, Mboni C, Saasa N, Sikasunge CS, Chelu C, Chisenga T, et al. Assessing trachoma elimination progress in districts with persistent trachoma, Western Province, Zambia. Int Health. 2025.

8.	MoH. Elimination of Neglected Tropical Diseases National Masterplan. Government of the Republic of Zambia. 2019.

9.	Mwale C, Mumbi W, Funjika M, Sokesi T, Silumesii A, Mulenga M, et al. Prevalence of Trachoma in 47 Administrative Districts of Zambia: Results of 32 Population-Based Prevalence Surveys. Ophthalmic Epidemiol. 2018;25(sup1):171-80.
